# Draft Genome Sequence of the Arsenic-Resistant Bacterium *Brevundimonas denitrificans* TAR-002^T^

**DOI:** 10.1128/genomeA.01326-17

**Published:** 2017-11-22

**Authors:** Taishi Tsubouchi, Yukihiro Kaneko

**Affiliations:** Department of Bacteriology, Graduate School of Medicine, Osaka City University, Abeno, Osaka, Japan

## Abstract

We report the 3.2-Mb draft genome sequence of *Brevundimonas denitrificans* strain TAR-002^T^, isolated from deep-sea floor sediment. The draft genome sequence of strain TAR-002^T^ consists of 3,231,216 bp in 44 contigs, with a G+C content of 68.47%, 3,866 potential coding sequences (CDSs), 3 rRNAs, and 45 tRNAs.

## GENOME ANNOUNCEMENT

The element arsenic exists widely in the environment (1). Organisms utilize arsenic as a trace element; however, arsenic is a toxic substance at high concentrations (2). While the organic arsenic compounds have low toxicity, inorganic arsenic compounds, such as arsenic acid, are extremely toxic; therefore, they are utilized as pesticides and wood preservatives (3). On the other hand, environmental pollution of soils and water quality caused by inorganic arsenic compounds has been a problem for a long time. Bioremediation, which uses the ability of microbes and plants to purify the contaminated environment caused by some heavy metals, such as cadmium, cobalt, and arsenic, has received much attention in recent years (4, 5). The Ars system in some microbes is well studied as an arsenic resistance mechanism-mediated arsenate reductase and arsenite transporter (6). Arsenic acid is reduced to arsenious acid by arsenate reductase; subsequently, arsenious acid is excreted by an arsenite transporter. Further, some microbes use the detoxification route, in which arsenious acid is converted to trimethylarsine by arsenite methyltransferase. The Ars system has been confirmed in *Escherichia coli*, *Staphylococcus aureus*, *Bacillus subtilis*, and so on (7–9). *Brevundimonas denitrificans* TAR-002^T^ (10), which is affiliated with the family *Caulobacteraceae*, shows arsenic resistance and seems to have some tolerance mechanism.

The draft genome sequencing of *B. denitrificans* TAR-002^T^ was performed on an Ion Torrent PGM sequencer (Life Technologies) equipped with a 318 chip. Data from the genomic DNA library contained 873,055 reads and 260,517,854 nucleotide bases, with an average read length of 298.34 bp using 400-base chemistry. Assembly using Newbler version 2.7 (Roche, Inc.) generated 44 contigs with maximum and minimum contig sizes of 240,832 bp and 568 bp, respectively.

The draft genome comprising 3,231,216 nucleotides was annotated with the help of the MetaGeneMark (11) and the Rapid Annotations using Subsystems Technology (RAST) server (12). These annotation results indicate that strain TAR-002^T^ possesses three different type of genes encoding arsenate reductase, which catalyzes the reduction of arsenic acid to arsenious acid. In the Ars system, arsenious acid is transported out of the cell via the ArsB protein (in some cases, with ArsA protein). ACR3, which exists in *B. denitrificans* TAR-002^T^, is a homolog of ArsB and is considered to function as an efflux pump. As for the detoxification metabolism, ArsM, which converts arsenious acid into trimethylarsine, is a volatile compound and exists in strain TAR-002^T^. In this genome analysis, it is clear that strain TAR-002^T^ possesses four* S*-adenosyl-methionine-dependent methyltransferase-coding genes. Moreover, ArsH, which is an organoarsenical oxidase that confers resistance to trivalent forms of organoarsenic compound, is also identified. On the other hand, the expression of these genes seems to be regulated by seven ArsR family transcriptional regulators, since one of them is clustered with these genes encoding ArsH, ACR3, and arsenate reductase.

### Accession number(s).

The draft genome sequence of *B. denitrificans* TAR-002^T^ reported in this paper has been deposited to the DDBJ/EMBL/GenBank under the accession no. BEWU00000000 (contig accession no. BEWU01000001 to BEWU01000044) in BioProject number PRJDB6380.
